# Oxidant resistant human apolipoprotein A-I functions similarly to the unmodified human isoform in delaying atherosclerosis progression and promoting atherosclerosis regression in hyperlipidemic mice

**DOI:** 10.1371/journal.pone.0259751

**Published:** 2022-02-04

**Authors:** Emmanuel Opoku, Stela Berisha, Gregory Brubaker, Peggy Robinet, Jonathan D. Smith

**Affiliations:** Department of Cardiovascular & Metabolic Sciences, Lerner Research Institute, Cleveland Clinic, Cleveland, Ohio, United States of America; University of Milano, ITALY

## Abstract

**Background:**

Transgenic overexpression of apolipoprotein A-I (apoA1) has been shown to delay atherosclerosis lesion progression and promote lesion regression in mouse models; however, apoA1 is subject to oxidation by myeloperoxidase (MPO) and loss of function. The activity of oxidant resistant human apoA1 was compared to unmodified human apoA1 in mouse models of atherosclerosis progression and regression.

**Methods and results:**

Human apoA1 and the MPO oxidant resistant 4WF isoform transgenic mice were bred to LDL receptor deficient (LDLr KO) mice and fed a western-type diet. High level expression of these human apoA1 isoforms did not lead to increased HDL-cholesterol levels on the LDLr KO background. In males and females, lesion progression was studied over time, and both apoA1 and 4WF transgenic mice vs. LDLr KO mice had significant and similar delayed lesion progression and reduced non-HDL cholesterol. Using time points with equivalent lesion areas, lesion regression was initiated by feeding the mice a low-fat control diet containing a microsomal triglyceride transfer protein inhibitor for 7 weeks. Lesions regressed more in the male apoA1 and 4WF transgenics vs. the LDLr KO, but the 4WF isoform was not superior to the unmodified isoform in promoting lesion regression.

**Conclusions:**

Both human apoA1 and the 4WF MPO oxidant resistant apoA1 isoform delayed lesion progression and promoted lesion regression in LDLr KO mice, with more pronounced effects in males than females; moreover, the 4WF isoform functioned similarly to the unmodified human apoA1 isoform.

## Introduction

Low levels of HDL-C do not appear to be directly causal for atherosclerosis in humans based on mendelian randomization genetic analysis [1]. In fact there appears to be a U-shaped relationship for the association of HDL-C with atherosclerotic disease, as those with the very highest HDL-C levels may have dysfunctional HDL, for example if the scavenger receptor B1 is defective, such that reverse cholesterol transport is impaired [2–4]. Thus, there is growing interest in HDL function, rather than its levels, as directly causal for protection from atherosclerosis [5,6]. One well-studied mechanism for HDL’s loss of function is through its oxidation by myeloperoxidase (MPO) [7], with about 1 in 5 HDL molecules recovered from human atheroma bearing the signature of this modification [8]. MPO modification of human apolipoprotein A-I (apoA1) leads to a loss of its ABCA1-dependent cholesterol acceptor activity when assayed on cholesterol labeled mouse RAW264.7 macrophages [7]. Furthermore, MPO oxidation of apoA1, compared to un-oxidized apoA1, destroys its ability to participate in reverse cholesterol transport and form HDL after injection into apoA1-deficient mice [9].

MPO can modify apoA1 on Met, Lys, Tyr, and Trp residues. We used site directed mutagenesis of recombinant human apoA1, and we found that altering all seven Tyr residues to Phe residues leads to a protein with excellent ABCA1-dependent cholesterol acceptor activity, which is still sensitive to MPO mediated loss of function [10]. Similarly, when the Met residues are altered to Val residues, the recombinant apoA1 is functional but also still sensitive to MPO-mediated loss of function [11]. Although, apoAI methionine oxidation appears to play a role in its MPO-mediated loss of lecithin:cholesterol acyltransferase activation [12]. However, upon making a recombinant apoA1 variant where all four Trp residues are replaced by Phe residues (4WF), the protein has excellent ABCA1-dependent cholesterol acceptor activity, but it resistant to MPO mediated loss of function [11]. Thus, apoA1 Trp oxidation, and not oxidation of other residues, is responsible for apoA1’s MPO-mediated loss of function as a cholesterol acceptor and HDL precursor.

We previously made 4WF transgenic mice (4WFtg) with equal apoA1 expression (~400 mg/dl and 270 mg/dl in males and females, respectively) compared to wild type human apoA1 transgenic mice (apoA1tg) [13]. The sex effect on human apoA1 levels in both transgenic lines was due to higher apoA1 production in males vs. females [13]. Both transgenic lines had ~70% decreased plasma levels of mouse apoA1 compared to non-transgenic mice [13], as previously reported [14–16]. Reverse cholesterol transport from injected cholesterol labeled macrophages to the feces was similar in both transgenic lines [13]. HDL purified from the apoA1tg and 4WFtg mice have similar total, ABCA1-dependent, and ABCA1-independet cholesterol acceptor activity in cultured cells [13]. However, upon ex-vivo modification of HDL from these two transgenic lines, we demonstrated that the wild type human apoA1 containing HDL loses all of its ABCA1-dependent cholesterol acceptor activity, while the 4WF containing HDL is partially resistant to MPO mediated loss of ABCA1-dependent cholesterol acceptor activity [13]. Here we extend our prior study of the apoA1tg and 4WFtg mice by breeding them on the LDLr-KO background, in order to study atherosclerosis progression and regression. We report that male and female western-type diet fed transgenic mice expressing either the 4WF or wild type apoA1 isoform on the LDLr KO background had lower levels of non-HDL-cholesterol and delayed aortic root lesion progression compared to control LDLr KO mice. Additionally, aortic root lesion regression, induced by feeding a regular rodent diet containing a microsomal triglyceride transport protein (MTP) inhibitor for seven weeks, was enhanced similarly by expression of either the wild type or 4WF apoA1 transgene, and was more pronounced in male mice.

## Materials and methods

### Ethics statement

Studies were conducted in conformity with the Public Health Service Policy on Humane Care and Use of Laboratory Animals and in accordance with protocols approved by the Cleveland Clinic Institutional Animal Care and Use Committee.

### Mice and diets

Mice were housed under standard conditions and given free access to food and water. Wild-type human apoA1 transgenic (apoA1tg) mice (C57BL/6-Tg (APOA1) 1Rub/J, #1927) were purchased from The Jackson Laboratory and backcrossed to C57BL/6 *Ldlr*^*-/-*^ (LDLr KO) mice (JAX #2207) to obtain apoA1tg heterozygotes LDLr KO mice. Further breeding of these mice to LDLr KO mice yielded ~50% LDLr KO and 50% LDLr KO with the apoA1tg (KO+A1tg) mice that were used in this study. The creation of 4WF transgenic mice on the C57BL/6 was previously described [13]. These mice were backcrossed to LDLr KO mice and a colony of mice homozygous for the 4WF transgene and LDLr KO (KO+4WFtg) was maintained and used in this study. For the progression study, LDLr KO, KO+apoA1tg, and KO+4WFtg mice were fed a western-type diet (WTD, Envigo # TD.88137), containing 21% fat and 0.2% cholesterol, upon weaning at 3 weeks of age for varying times as indicated. For the regression study, different genotypes at the specified ages were used to begin with matched baseline lesion areas, and mice were switched to a low fat control diet (Envigo 2018) containing the MTP inhibitor BMS 212122 (kindly supplied by Bristol Myers Squibb) at 25 mg/kg diet (Envigo TD.140406) for 7 weeks before sacrifice. For lesion progression and regression studies, a power analysis was performed (GraphPad InStat software). We determined that N = 10 per genotype per sex per time point yielded 80% power to see a 33% effect on lesion area in a 2-tailed analysis at a p-value of 0.05, assuming a coefficient of variation of 25%. Thus, we attempted to achieve N = 10 per time point, although due to poor breeding or loss of mice we did not achieve this number for all conditions.

### Plasma HDL-C, non-HDL-C, total cholesterol, and apoA1 quantification

Plasma HDL-C was isolated from non-HDL cholesterol by a two-step procedure that we use for all hyperlipidemic mouse plasma samples. The first step involves spinning up non-HDL cholesterol. 40 μl each of mouse plasma and KBr solution at 1.12 g/ml were added to a polycarbonate micro-ultracentrifuge tube (Beckman Coulter # 343775). After mixing, the tubes were centrifuged at 70,000 rpm for 16 hours in a S1000AT3 rotor in an Optima XPN-100 micro-ultracentrifuge (Beckman Coulter). The bottom 40 μl, containing HDL, was removed to a clean microfuge tube and 4.4 μl of HDL reagent (StanBio # 0599020) was added, vortexed, and spun to precipitate any remaining apoB-containing lipoproteins. The total cholesterol content in 10 μl of plasma (diluted 1:20) and HDL were determined using a colorimetric cholesterol assay (StanBio, 1010430), with HDL-C multiplied by 1.1 to account for dilution with the HDL reagent. Non-HDL-C was calculated as total cholesterol minus HDL-C. Human apoA1 levels in mouse plasma were determined by an automated clinical immunoturbidimetric assay (APOAT ver.2, Roche).

### Lesion quantification

Mice were euthanized by carbon dioxide inhalation, and perfused with 10 ml PBS. Formalin fixed hearts were sectioned through the aortic root, stained with Oil Red O and hematoxylin, and lesion areas quantified, as previously described [17].

### Size exclusion chromatography lipoprotein profile

Fast performance liquid chromatography (FPLC) was performed using pooled plasma from 5 mice (250 μL loop at 0.5 mL/min) on a Superose 6 10/300 GL column (GE Lifesciences). 0.5 mL fractions were collected and total cholesterol for each fraction was measured as described above.

### Statistical analyses

Statistics were performed using Prism software v.9 (GraphPad). All data was tested for normality and passed the Shapiro-Wilk or KS test. Graphs show means ± S.D. The progression time course data was analyzed by 2-way ANOVA using age and genotypes as sources of variation, with Tukey’s multiple comparison test for genotype effects. The regression data was analyzed by ANOVA, with Sidak’s multiple comparison tests for 9 selected pairwise comparisons (3 genotypes compared to each other at baseline, 3 genotypes compared to each other at regression, and 3 baseline-regression comparisons within each genotype).

## Results

### Lesion progression study

**Fig 1:**
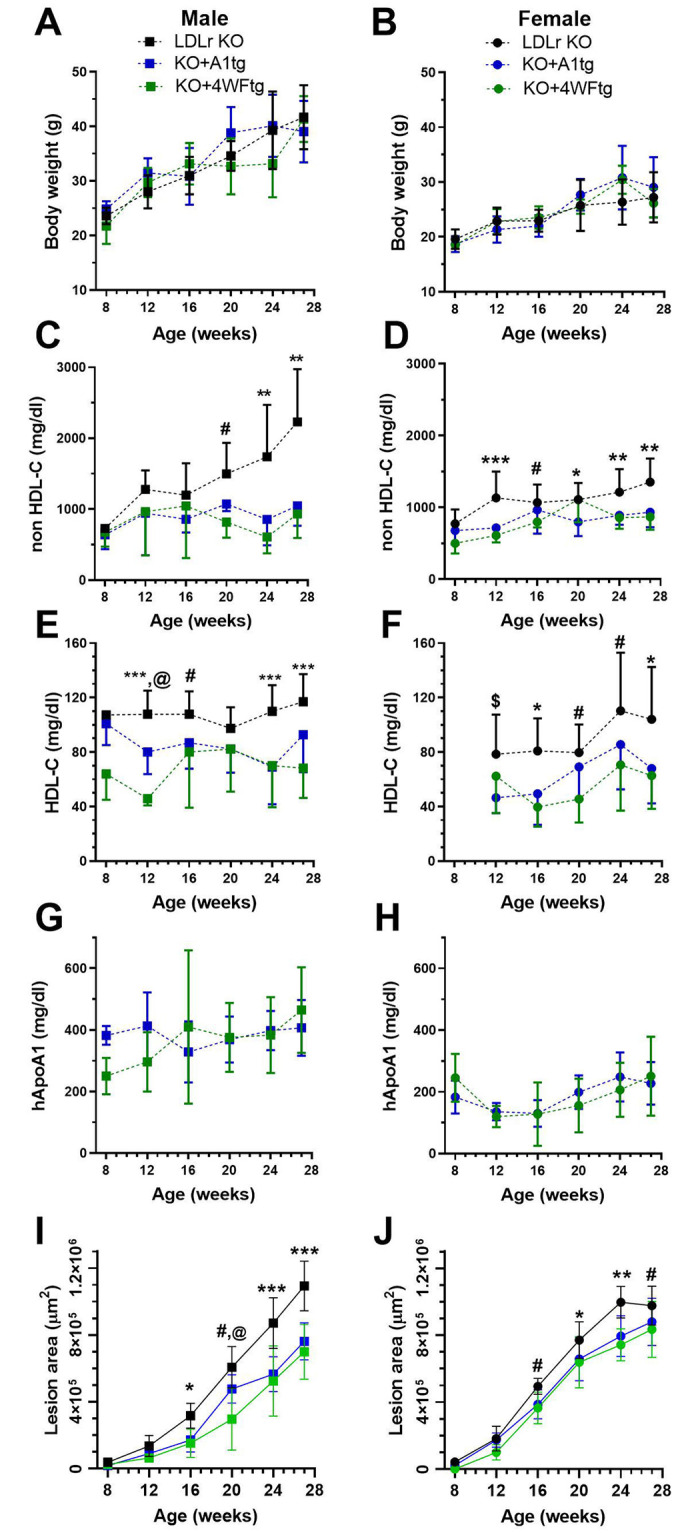
Lesion progression time course in male (square symbols, left side) and female (round symbols, right side) mice. **A,B**. Body weights during the progression time course. **C,D**. Non-HDL-C levels. **E,F**. HDL-C levels. **G,H**. human apoA1 levels. **I,J**. Aortic root lesion areas. For all panels, black symbols are LDLr KO mice, blue symbols are LDLr KO expressing the wild type human apoA1 transgene (KO+A1tg); and green symbols are LDLr KO expressing the 4WF apoA1 transgene (KO+4WFtg). Mean±SD; two way ANOVA p-values: #, p<0.05 for LDLr KO vs. KO+4WFtg; @, p<0.05 for KO+A1tg vs. KO+4WFtg; $, p<0.05 LDLr KO vs. KO+A1tg; *, p<0.05 for LDLr KO vs. both transgenic isoforms; **, p<0.01 for LDLr KO vs. both transgenic isoforms; ***, p<0.001 for LDLr KO vs. both transgenic isoforms.

We performed a time course for lesion progression in male and female LDLr KO, KO+A1tg, and KO+4WFtg mice that were weaned on a WTD, sacrificing different cohorts at 8, 12, 16, 20, 24, and 27 weeks of age. Body weights increased with age, more so in the male mice, with no genotype effect (Fig 1A and 1B). Although non-HDL-C levels started out equivalently at 8 weeks of age in both sexes, upon aging the non-HDL-C levels were significantly higher in the LDLr KO mice vs. both transgenic lines, particularly at the 24 and 27 week time points, and more pronounced in males (Fig 1C and 1D). We previously found that the expression of the human apoA1 or 4WF-apoA1 transgenes in mice, which were not on the LDLr KO background, led to large increases in HDL-C, more so in male than female mice [13]. Unexpectedly, in the current study, there was a non-significant trend for lower HDL-C in transgenic males vs. LDLr KO mice, and a significant lowering of HDL-C in the female transgenic vs. LDLr KO mice (Fig 1E and 1F). Human apoA1 levels were robust and did not change over time in both male and female transgenic lines, ~ 200–400 mg/dl and ~100–200 mg/dl, respectively (Fig 1G and 1H). Aortic root lesions progressed over time with the 2-way ANOVA showing significant age, genotype, and age-genotype interaction effects in both sexes. At 16 weeks of age, lesions were larger in LDLr KO vs. the KO+A1tg (45% smaller) and KO+4WFtg (52% smaller) mice, with the effect gaining in significance with age, more so in males vs. females (Fig 1I and 1J). At 24 and 27 weeks of age, this effect was highly significant in males (p<0.001 after adjustment for multiple testing). For example, 24-week old male KO+A1tg and KO+4WFtg mice had 35% and 40% smaller lesions, respectively, than the LDLr KO males.

### Lesion regression study

**Fig 2:**
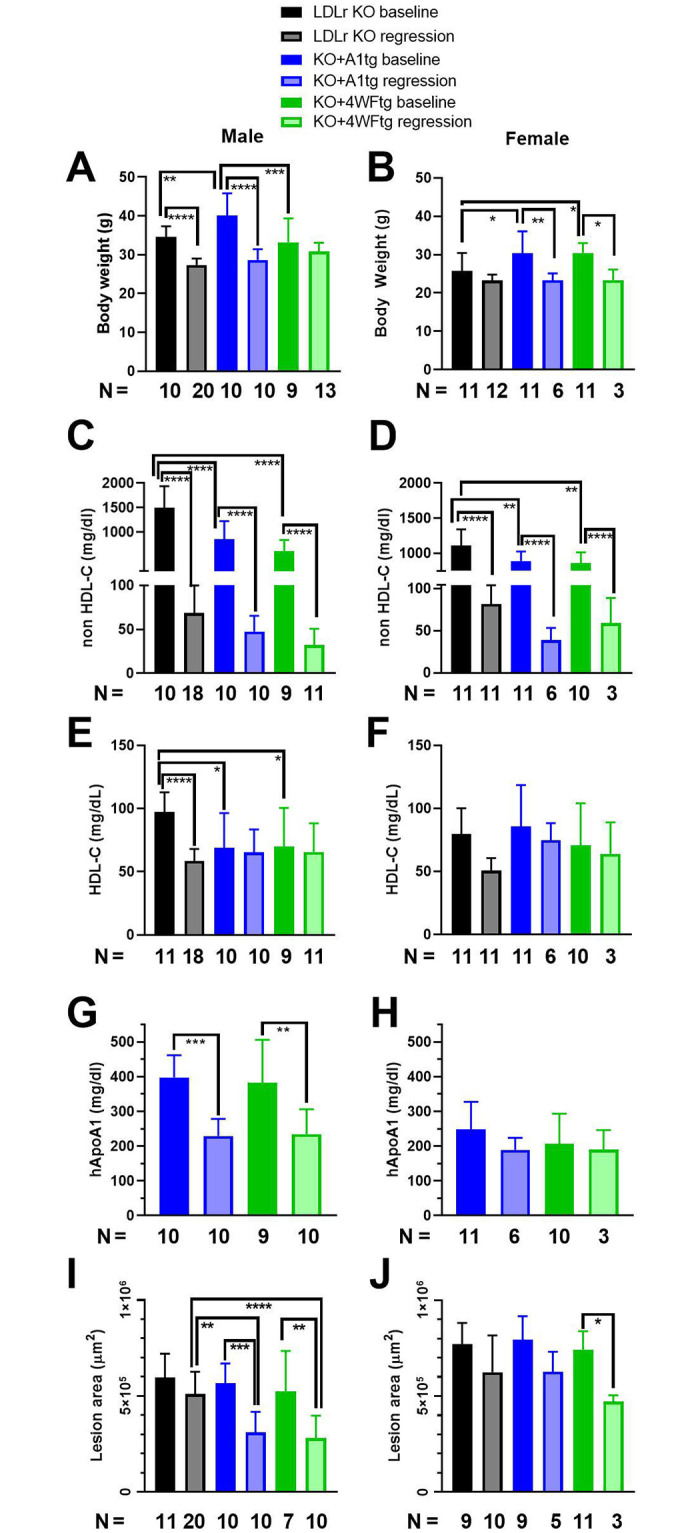
Lesion regression (unsaturated colors) from baseline (saturated colors) in male (left side) and female (right side) mice. **A,B**. Body weights at baseline and after seven weeks on the regression diet. **C,D**. Non-HDL-C levels. **E,F**. HDL-C levels. **G,H**. human apoA1 levels. **I,J**. Aortic root lesion areas. For all panels, black bars are LDLr KO mice, blue bars are LDLr KO expressing the wild type human apoA1 transgene, and green bars are LDLr KO expressing the 4WF human apoA1 transgene. Mean±SD with the number of mice for each column at the bottom; ANOVA Tukey posttest p-values: *, p<0.05; **, p<0.01; ***, p<0.001; and ****. p<0.0001. The N under the bars represents the number of mice used for each measurement. The numbers may vary if insufficient plasma was obtained for cholesterol measurements or the aortic root lesion sectioning was not perpendicular to the aortic root.

**Fig 3:**
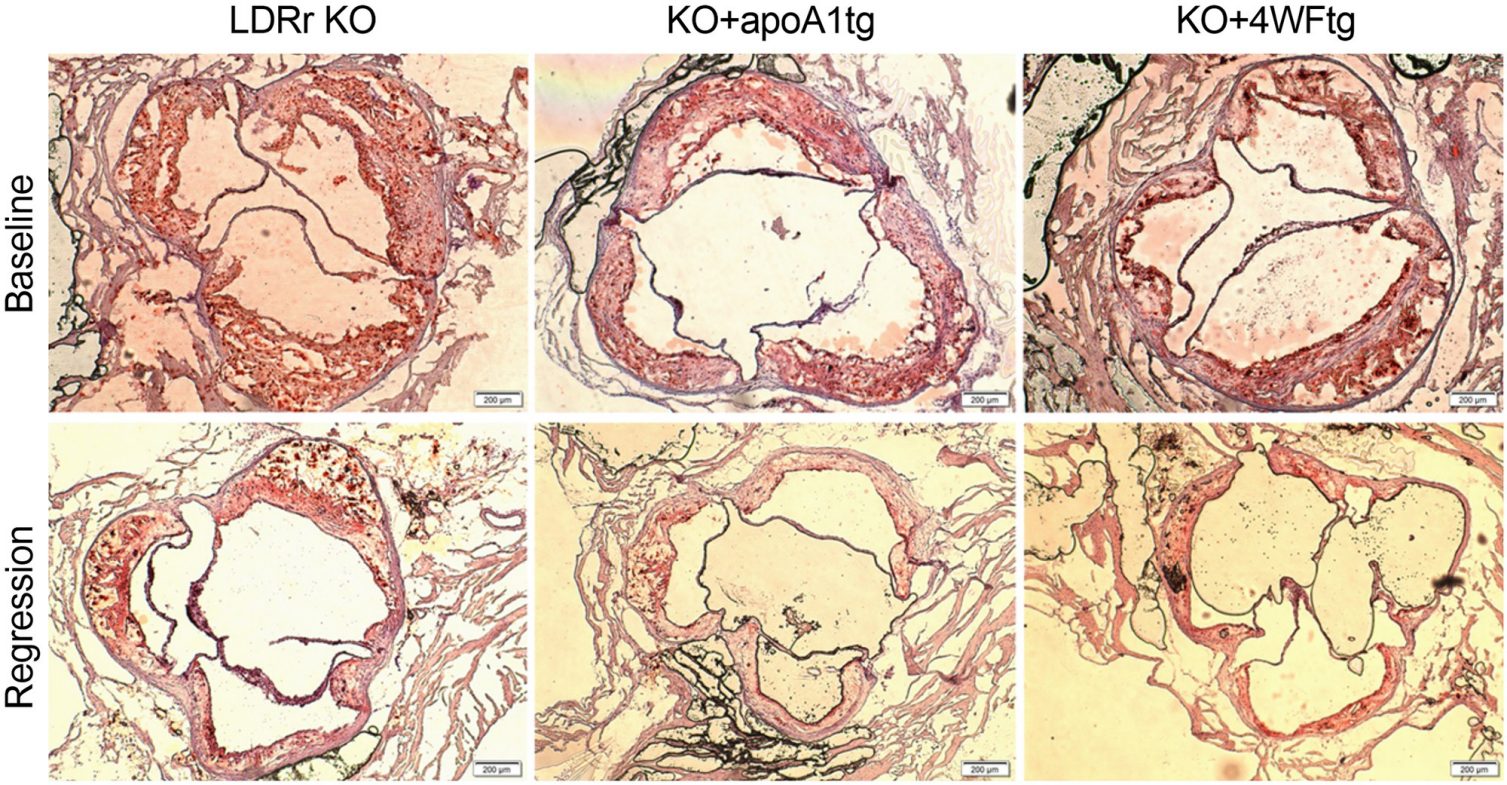
Aortic root lesions. Representative baseline (top) and regression (bottom) aortic root lesions from male mice of the LDLr KO (left), KO+apoA1tg (center), and KO+4WFtg (right) genotypes stained with Oil Red O and hematoxylin (4x objective lens, 200 μm scale bar).

The baseline time point for the regression study was chosen based on the progression time course so that the aortic lesion areas were similar in the LDLr KO (20 weeks) and the two apoA1 transgenic lines (24 weeks). Regression was initiated by switching to a low fat regular rodent diet including the MTP inhibitor BMS 212122, as previously described [18], and maintaining the mice on this diet for seven weeks. The regression diet generally led to significant decreases in body weights in both sexes except for the male KO+4WFtg and female LDLr KO mice, which displayed nonsignificant trends (Fig 2A and 2B). The baseline non-HDL-C levels were significantly lower in the two transgenic lines vs. LDLr KO in both sexes. The regression diet led to large and significant decreases in non-HDL-C, achieving values <100 mg/dl in all groups (Fig 2C and 2D). The baseline HDL-C levels were lower in males of both transgenic lines vs. LDLr KO, and the regression diet significantly lowered HDL-C only in the male LDLr KO mice (Fig 2E). There were no significant differences in HDL-C in any of the female groups (Fig 2F). We previously found that human apoA1 levels are higher in males vs. females for both A1tg and 4WF transgenic lines that were not on the LDLr KO background [13]. We observed the same phenotype on the LDLr KO background, with males transgenics having higher human apoA1 levels vs. females (p<0.01, Fig 2G and 2H). The regression diet significantly lowered human apoA1 levels in males, but not females, of both transgenic lines (Fig 2G and 2H), despite no significant lowering in their HDL-C levels. At baseline, the lesions in the male mice were all similar; and, the regression regimen led to significant decreases in lesion areas in both the KO+A1tg (45% smaller) and KO+4WFtg (47% smaller), but only a trend in the same direction in the LDLr KO males (15% smaller) (Fig 2I). At the end of the regression period, the lesion areas were significantly smaller in the male KO+A1tg (39% smaller) and KO+4WFtg (45% smaller) vs. LDLr KO (p<0.001 and p<0.0001, respectively, Fig 2I). Examples of baseline and regression aortic root lesion micrographs for the three genotypes of male mice are shown in Fig 3. Regression in the female mice was not as pronounced despite the robust lowering in non-HDL-C; and, the only significant decrease was in the regression vs. baseline lesion area for the KO+4WFtg females (36% decrease) (Fig 2J). However, the regression lesion areas were not significantly different among the three female genotypes (Fig 2J).

**Fig 4:**
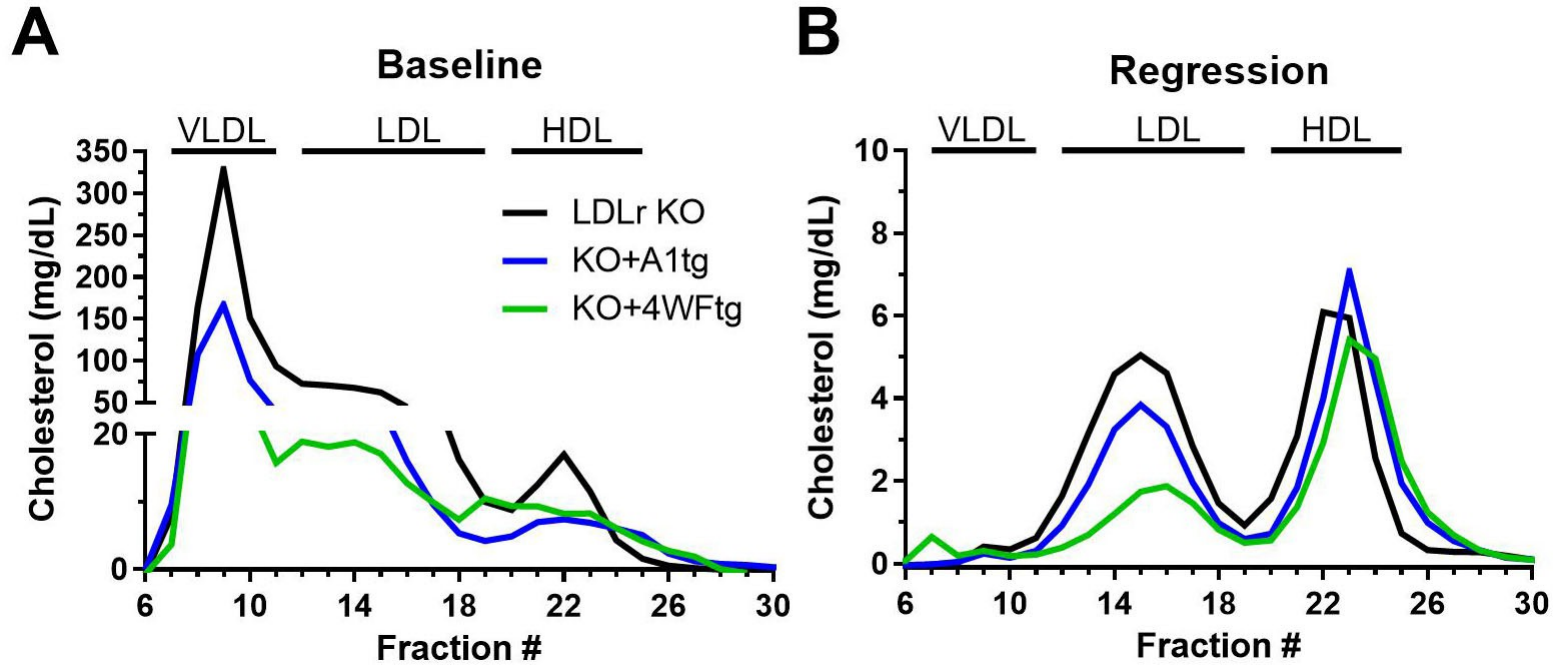
FPLC lipoprotein profile of male mice at the baseline time point on the western-type diet (A) and 7 weeks after starting the regression diet (B). Cholesterol concentrations in each 0.5 ml fraction shown, note the scale difference between the progression and regression panels. Black line, LDLr KO mice; blue line, LDLr KO expressing the wild type human apoA1 transgene; and green line, LDLr KO expressing the 4WF human apoA1 transgene.

We performed FPLC lipoprotein analysis for the males, which had more robust lesion regression, to confirm the genotype effects on the lipoprotein profiles (Fig 4). At baseline, on the western-type diet, most of the cholesterol was found in the large VLDL and LDL size range, with lower levels of VLDL and LDL size range observed in the KO+A1tg and KO+4WF transgenics vs. LDLr KO, with this effect on non-HDL-C appearing even more pronounced than the ultracentrifuge assessment (compare Fig 4A with Fig 2C). Switching to the regression diet led to dramatic shifts in the lipoprotein profile for all three genotypes, with the VLDL peak dramatically reduced, while retaining the effect of the apoA1 and 4WF transgenes on lowering the non-HDL-C levels (Figs 2C and 4B). The HDL-C fractions appeared similar in all three genotypes, despite some changes observed in the biochemical assay (Figs 2E and 4B).

## Discussion

We compared the MPO oxidation resistant 4WF apoA1 isoform vs. the wild type human apoA1 in the context of the LDLr KO mouse model of atherosclerosis progression, and a diet switch plus MTP inhibitor atherosclerosis regression model, developed by Fisher and colleagues [18]. Our prior work characterized the 4WFtg and A1tg mouse lines, showing similar human apoA1 and HDL-C levels in these strains, and that both strains had higher HDL-C vs. non-transgenic control mice. Thus, we were perplexed by our current finding that despite robust human apoA1 levels, the HDL-C levels trended lower during the progression study for both KO+apoA1tg and KO+4WFtg lines vs. LDLr KO mice (Fig 1E and 1F). The lower HDL-C trend was also observed in the baseline FPLC profile (Fig 4A). The lower HDL-C might be due to altered particle composition, and there does appear to be elevated levels of small, protein-enriched, HDL in fractions 25–27 in the FPLC profiles from the two apoA1 transgenic lines vs. the LDLr KO line. Previously, more lipid-poor pre-beta-migrating apoA1 was observed in human apoA1 transgenic mice on the LDLr KO background vs. control LDLr KO mice [19], and this lack of HDL formation or maturation might also be a factor in lower HDL-C in the apoA1 transgenics.

The other effect of both apoA1 isoform transgenes on plasma lipoproteins was the highly significant decrease in non-HDL cholesterol seen in both sexes (Fig 2C and 2D), which was also observed previously [19]. This could be due to lower production rate or a higher turnover rate, and would take further study to unravel.

In order to evaluate the effect of the two apoA1 isoforms on regression, we used the diet switch plus MTP inhibitor model as a simple method to ameliorate the hypercholesterolemia in atherosclerosis prone mice. The original paper describing this regression model maintained mice on the regular diet plus MTP inhibitor diet for three weeks, which was insufficient to significantly decrease the plaque area, although the oil red O staining fraction of the plaque was significantly reduced [18]. Thus, we decided to extend the regression period to seven weeks, which was sufficient to see significant reductions in plaque areas, vs. baseline, in both apoA1 transgenic lines in males and for the LDLr+4WF transgenic females (Fig 2I and 2J).

We observed many sex differences in the current study and we reported all measures for both sexes separately, as is recommended [20]. We previously found that human apoA1 levels were higher in male vs. female transgenic mice, which was due to higher production rates in turnover studies [13]. Here we found a similar result with male vs. female transgenics having increased non-HDL-C and human apoA1 levels when fed the WTD (Fig 2C, 2D, 2G and 2H). As usual for mice aortic root lesions [17], females at baseline had larger lesions than males (F 2I, J). The reason for more pronounced regression in male than female mice is not known. It might be due to starting with smaller lesions that are easier to remodel, but the sex difference in lesion regression is not easily explained by either HDL-C or non-HDL-C levels, as they are similar in both sexes after switching to the regression diet. Another possibility is that a function of HDL, such as reverse cholesterol transport or anti-inflammatory activity, is stronger in the male vs. female apoA1 transgenic mouse lines. Additional studies would be needed to determine the mechanisms responsible for increased atherosclerosis regression in males vs. females for these mice.

In conclusion, the MPO resistant apoA1 4WF isoform behaved similarly, and was not superior, to the wild type human apoA1 isoform in promoting lesion regression in LDLr KO mice after switching to a regression diet. One potential reason for the non-superiority of the 4WF isoform is that mice express much lower levels of MPO compared to humans [21]. Thus, in a high MPO context, the 4WF isoform might show an advantage over the wild type isoform.

## Supporting information

S1 File Data used for the graphs in Figs 1, 2 and 4, including aortic lesion areas for each section quantified. All data arranged by sex, genotype, and time point.
(XLSX)
